# Combining Deep Learning Mechanisms to Predict Interests from Gaze

**DOI:** 10.3390/s26102975

**Published:** 2026-05-09

**Authors:** Aoba Izumi, Fumiko Harada, Hiromitsu Shimakawa

**Affiliations:** College of Information Science and Enginnering, Ritsumeikan University, Osaka 567-8570, Japan; aoba250cc@de.is.ritsumei.ac.jp (A.I.); harada@fc.ritsumei.ac.jp (F.H.)

**Keywords:** gaze, interest, saliency map, deep learning, transformer

## Abstract

This paper proposes a model to predict users’ personal interests from their gaze. Current image processing technologies enable us to identify each user’s gaze and pupil diameter. However, they cannot identify user interests. The model uses deep learning technologies. It consists of mechanisms to handle the two kinds of gaze: one specific to individuals and the other common to many people. Its training method utilizes the human property that user interests in objects in vision affect their gaze. Since it is known that pupil diameters increase when users view what they are interested in, we can train the model to predict users’ personal interests using the pupil diameters as labels. Notably, the paper not only proposes a model structure to predict personal interests but also examines the process of the modification of parameters of the model to reveal requisites to predict personal interests from gaze. The method enables us to provide personalized information to each user, such as recommendations in VoD services, advertisements during e-shopping, plate annotations in restaurant menus, and so on.

## 1. Introduction

Human interests vary from person to person. If we could predict what a person is interested in, there are a variety of potential applications, such as displaying advertisements during e-shopping for individual customers or presenting annotations for cuisines on restaurant menus based on personal preferences. The applications include targeted advertising, which attracts interest in appropriate products based on the user’s interests. It is based on the idea that the users’ interests are expressed in the content they search for and watch. Data obtained from the users brings the product recommendations [1,2,3]. These approaches have been actively researched in recommendation systems.

Collaborative filtering has achieved the greatest results in this field. This method assumes that user preferences are consistent across many users. It predicts the interests of a specific user based on the similarity with many users. Leveraging the interests shared by many users, collaborative filtering can recommend optimal products and advertisements. It enriches their lives.

However, collaborative filtering only works well when many users are interested in objects with known characteristics. It also assumes the user whose interests we want to predict shares common interests with many users. The issue of interests common to users versus user-specific interests has also been pointed out in recommendations in another field. The work [4] demonstrates that news recommendations involve both user-dependent interest effects and user-independent timeliness effects. Collaborative filtering provides no benefit to grey sheep users, whose interests are only partially consistent with those of other users and have low correlation coefficients with almost all other users. The interests of users who have unique characteristics within a population are difficult to predict. It is known as the grey sheep problem [5]. Since recommender systems should benefit all users, there have been many attempts to predict the interests of grey sheep users [6,7].

To identify the unique interests of a particular individual, it is meaningless to examine only characteristics common to a large number of users. It is necessary to predict items that will be of interest to each user based on their unique characteristics. To do this, we need indices whose value changes depending on the interests of each user. Such indices have only been calculated for specific media, such as [1,2,3]. If we could obtain an index that can identify users’ interests from their everyday activities, the power of recommendation systems could be extended to all fields.

A person’s gaze contains many meanings. Interest is one of them. It is known that a person’s gaze changes when what they are looking at is interesting to them. Because gaze directly expresses individual interests, it not only expresses the factors of persons’ interests but also reveals their characteristics. The work [8] reports that a saliency map demonstrates human attention during e-shopping. It also shows that attention is influenced not only by visual characteristics such as color, contrast, and brightness but also by cognitive processes such as expectations and prior knowledge. The survey of deep learning-based saliency detection methods [9] shows that saliency maps in e-commerce are useful to lead customers’ attention to detailed product information, which contributes to improving visual search, personalized recommendations, and user engagement. Furthermore, people would look at objects of interest. It does not place any extra burden on them. Gaze is a suitable indicator for predicting interests.

If we could predict an individual’s interests from their gaze, we could solve the grey sheep problem. However, to make the predictions, we need to use gaze information collected from individuals to train personal AI models. It requires a long time because the gaze information must be repeatedly presented to the model for training. A low-cost method is needed to build a model that can predict an individual’s interests from their gaze. It is necessary to clarify the structure of a prediction model that can be trained efficiently.

This study proposes a model structure for predicting individual interests from gaze. To achieve interest prediction at low cost, it first shows that individual interests affect the distribution of gaze. Second, to efficiently predict individual interests from gaze, the study proposes a model that combines a mechanism for predicting gazes common to many people with one for extracting individual interests. Both of the mechanisms are implemented using deep learning models. Finally, experiments reveal how parameters of the mechanisms are modified during training. They also demonstrate that parameters of a pre-trained mechanism for predicting gaze common to many people improve the training efficiency of extracting individual interests.

The proposed model enables us to know what interested individuals look at. Due to the model, the recommendations of products and advertisements benefit all users. Furthermore, the learning process elucidated in this study will lead to ideas for making individual interest predictions more efficient.

## 2. Gaze and Interests

### 2.1. Saliency Map

Human gaze moves continuously depending on the purpose. When there is no target to look at, the gaze moves randomly, which means random gaze movements do not indicate interest. On the other hand, when humans are interested, they will fix their gaze on the object of interest [10]. For a given task, gaze changes to complete it [11]. It comes from the dynamic switching of the attention focus. Experiments with paintings [12] have revealed that gaze movements of persons interested in paintings differ from those of people with no interest in paintings, though they engage in the same task to view the paintings.

Saliency maps show which parts of an image are attracting attention. Let us consider periodically observing the points a person looks at for a specific image. A saliency map visualizes image areas where the person tends to put gaze by displaying a scatter of points indicating observed gaze on top of the image. Saliency maps are used in UI analysis [13] and clinical experiments using eye tracking [14], taking advantage of their properties. This study aims to predict image areas in which a person is interested. If gazes are focused on an object, there is a high possibility that the person is interested in the object. The study uses a saliency map to visualize differences in individual interests.

### 2.2. Pupil Diameter

The pupil diameters indicate whether a person is interested in something. It is known that the size of people’s pupils changes depending on their interests. When looking at something that interests them, their pupils expand. On the other hand, pupils get smaller when they see what does not interest them—their pupils constrict [15]. Observation of these changes in the pupils provides us with information about interests that simple staring fails to present.

Eye tracking is a technology for observing gaze. It uses a specialized sensor called an eye tracker to record where a person is looking. Many works using eye tracking have been actively studied in recent years. It is used in a wide range of fields, including psychology and HCI [16].

To generate a saliency map, eye tracking is used to observe where people are looking within an image [17,18]. Some eye trackers can observe not only the points people are looking at within an image, but also the pupil diameter. However, eye trackers are expensive because they measure gaze and pupil diameter simultaneously. Furthermore, note that targets of interest are identified from pupil diameter, not from the saliency map.

### 2.3. Predicting Gaze for Each User

Gaze changes depending on an individual’s interests. Even when viewing the same image under the same conditions, the objects that attract attention within the image would differ from person to person. An individual’s saliency map differs from a saliency map that aggregates the gazes of many people. A saliency map that predicts an individual’s gaze is referred to as a personalized saliency map (PSM), while a saliency map that aggregates the gazes of many people is referred to as a universal saliency map (USM).

To create a USM, we need an image and the distribution of gazes toward it. To predict the USM, a deep learning method trains a model using the difference of the model output from the distribution of gaze that many people actually put on the image. It optimizes the model to minimize the difference. Deep learning models with convolutional layers are often used to process images. Among them, many industrial examples adopt VGG models, which combine convolutional layers with ReLU functions [19].

To predict gaze for each individual, it is necessary to incorporate individual features into the gaze prediction model. To extract individual features, one work labels what each individual is looking at [20], while another provides a user embedding layer using PSM [21]. These predict gaze for each individual by directly incorporating individual features as numerical data.

Another work uses BART for user encoding [22]. It utilizes the ability to calculate relationships between data, a property of the attention mechanism. PSM prediction requires calculating the relationship between each individual’s interest and features within the image. The encoder layer of a Transformer model represents the relationship between them as keys and values. The decoder emphasizes the query based on the keys and values passed from the encoder layer [23]. The emphasis of features within the image with an individual’s interest vector enables us to build a model that predicts gaze for each individual. The method of using the attention mechanism to represent differences between individuals has also been applied to fields other than saliency map prediction [24,25].

### 2.4. Large-Scale Data for Predicting Gaze

To predict individual gaze, a predictive model must be able to identify areas in an image where people are likely to gaze. Interesting factors must have sufficient characteristics to attract attention within the image. If there is no object in the image that attracts attention, gaze will likely be dispersed over a wide area. To reflect interest in a human saliency map, we need a mechanism that can predict factors that attract general attention.

Large-scale saliency map data is essential for building a gaze prediction model using deep learning. However, the amount of data required for deep learning is enormous. It is impractical to recruit subjects and create a saliency map dataset from scratch. It is a common way to use a saliency map dataset created in previous works to build a model for predicting the gaze of a large number of people.

One well-known dataset is the SALICON dataset. Participants use a mouse to indicate the object they are looking at in an image displayed on a personal computer. The data is collected on a large scale via a server, resulting in the SALICON dataset [26]. The gaze distribution in the dataset has been proven to be as accurate as a saliency map created using gaze data. The dataset adequately represents human gaze. Furthermore, the dataset contains 10,000 training images and 5000 validation images, making it a sufficient amount for use as a deep learning dataset. Due to its large amount of data, it can be used to build a saliency map prediction model [27]. A gaze prediction model using the dataset guarantees the ability to predict general human gaze.

## 3. Gaze Model Reflecting Interest of Each User

### 3.1. Research Questions

This paper addresses the following research questions.

- Do interests affect persons’ gaze?
- Can we build a model that predicts the interests of persons with their gaze?
- What kinds of process does the model learn to predict interests with gaze?

Though the first would be right intuitively, we should confirm it through an experiment. The experiment would reveal the validity of the model configuration we assume in the second question. Once we have built the model, we can investigate the learning process of the model.

Interests vary with individuals. The model has to be trained for each user, which means it costs much to provide personal service. The investigation of the learning process would provide a way to solve the cost problem.

To address the questions, this paper handles two kinds of models to predict gaze for a specific image. One uses gaze data collected from a large number of people to predict a universal saliency map (USM), which indicates image parts where many people’s gazes are likely to be focused. The other predicts a personal saliency map (PSM), which indicates the image parts where an individual’s gaze is likely to be focused.

**Figure 1:**
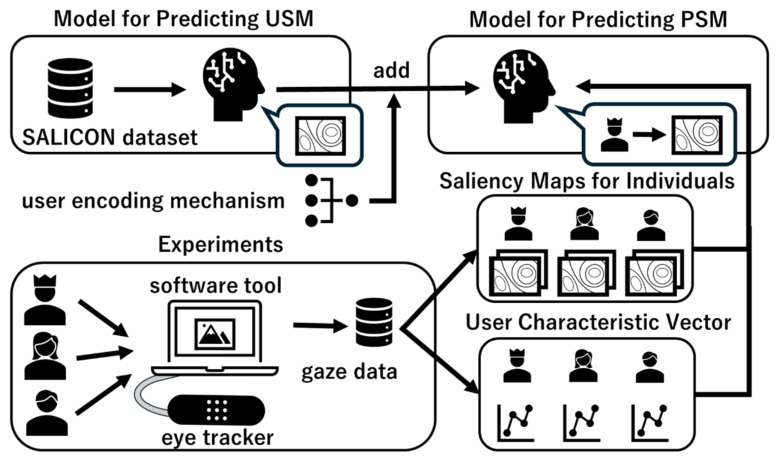
Outline of building models for predicting USM and PSM.

The models are obtained according to the procedure shown in Figure 1. A model predicting USM is trained with the SALICON dataset, while a model predicting PSM is trained with individual saliency maps and user characteristic vectors collected in experiments. In the experiments, a variety of images are presented to users to collect their gaze data using an eye tracker. A proprietary software tool standardizes the viewing and rest time for every user. Individual saliency maps are generated from the gaze data. Pupil diameter is collected along with gaze. An anomaly test on each user’s pupil diameter extracts the user’s interests as a user characteristic vector. The details of each process are explained in the following subsections.

### 3.2. Model for Predicting USM

A model with complex mechanisms might make it unclear which configuration is effective for gaze prediction. This study builds a simple model.

**Figure 2:**
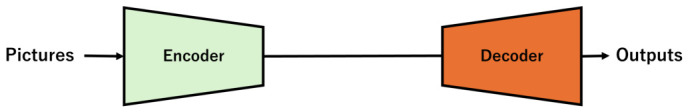
Model for predicting USM.

Figure 2 shows the general gaze prediction model proposed in the study. The model is based on SalGAN, a well-known saliency map prediction model [28]. Since it follows the GAN mechanism, it consists of a generator and a discriminator. When predicting gaze, the study does not use a discriminator. It uses only the generator of a saliency map. The generator uses a VGG16 as its encoder. Its output is fed to the decoder to generate a USM. The model configuration is shown in Table 1 for the encoder and Table 2 for the decoder.

SalGAN uses a sigmoid function as the activation function for the final layer to use BCE (Binary Cross Entropy) as the loss function. However, this study uses a ReLU function. It results in a non-negative output. It allows us to use mean squared error as the loss function, which makes the accuracy of the generated saliency map easier to interpret intuitively.

In this study, to make the calculation light, the input image is compressed to 1/4 of its size. SalGAN can achieve sufficient accuracy even with input images compressed to 1/4. There is no impact on prediction results.

### 3.3. Collection of Saliency Maps from Individuals

**Figure 3:**
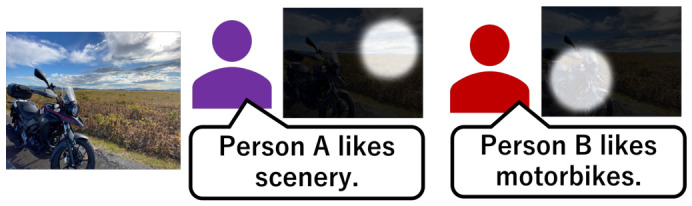
Saliency maps dependent on individuals.

To predict the interests of each individual, it is desirable that when persons view a specific image, the location where their gaze is focused differs depending on their interests. An example is shown in Figure 3. Suppose an image in which a rural landscape occupies the right half, while a motorcycle occupies the left half. Person A, who likes landscape paintings, primarily fixates on the right half. On the other hand, person B’s gaze will likely be focused on the motorcycle on the left half. For gazes to differ from person to person, the image should present various objects.

Based on the assumption that human gaze is drawn to areas within an image that contain meaning or context, the SALICON dataset is composed of images selected from the MS COCO dataset [29]. Each image in the dataset contains a variety of interesting objects.

Predicting a saliency map requires human gaze information collected for each image. Following the method used to create the SALICON dataset, gaze data collection using an eye tracker requires alternating between displaying an image for a certain period of time to have a person observe it and taking a break for a certain period of time. If the user is required to change the image, the operation increases the cognitive load on the person to introduce noise into the gaze information [30].

This study prepares a tool to collect gaze information. The data items collected per unit time by the gaze collection tool are shown in Table 3. Once launched, it will display an image that covers the entire screen. It allows the entire PC screen to be used for gaze collection, reducing noise during collection. After launching, the tool automatically switches between image presentation and rest phases. The image changes when switching from rest to image presentation.

### 3.4. Generating Saliency Maps for Individuals

The tool periodically collects the coordinates of the points on the image that the person is looking at. For each coordinate in the image, it calculates the number of times the gaze is fixed on the point within a certain period. The number indicates where the user is looking and for how long. The saliency map is created by applying a Gaussian filter to the number and representing it as a heat map between 0 and 1.

When gazes are concentrated around a specific image coordinate, the peak of the heat map will be higher than other image coordinates, emphasizing that gazes are focused on that object. Conversely, in areas where gazes are not concentrated, there will be no peak on the heat map at all.

Generating such a saliency map, we can express where and how much the person is looking as the probability of gaze appearing for each image coordinate, rather than as a simple set of gaze coordinates.

### 3.5. User Characteristic Vector

This study uses pupil diameter as an indicator of interest. However, just like changes in gaze, pupil diameter also changes from moment to moment. A simple comparison of pupil diameter between the previous and current times is insufficient to determine whether pupil diameter has changed due to interest. Furthermore, pupil diameter differs from person to person due to physical characteristics. Suppose we determine that interest exists when the change in pupil diameter exceeds a certain threshold. However, if a uniform threshold is applied to multiple persons, the influence of their unique pupil diameter size will make it impossible to accurately determine whether each of them is interested.

Suppose we record the change in pupil diameter over *t* seconds while a user is viewing an image. If a user views *M* images, we can observe the change in pupil diameter for each subject over tM seconds. If pupil diameter follows a Gaussian distribution, the histogram of pupil diameter over tM seconds should be bell-shaped, centered on the mean value, for each subject. If no changes in interest occur, pupil diameter will fluctuate around the mean value. On the other hand, if the pupil expands or contracts due to interest while viewing a particular image, the probability of large or small pupil diameter values increases. Therefore, this study calculates the Mahalanobis distance of the acquired pupil diameter for each user to perform an anomaly test. The significance level used in the test determines a threshold for pupil diameters that are larger or smaller than normal. Based on the threshold, we can extract images for which the pupil diameter enlarges for each subject.

For the experimental dataset *D*, let $D_{nmt}$ be the pupil diameter at time *t* when person $P_{n}$ observes image $S_{m}$. Using the function $f_{p}$, $D_{nmt}$ is defined as follows:

$$D_{nmt}=f_{p}\left(t,P_{n},S_{m}\right)$$

The threshold value obtained by the abnormality test is *A*, which is set to 1 if the pupil diameter is enlarged and 0 otherwise. In this case, whether person $P_{n}$ is interested in image $S_{m}$ is defined using function $f_{i}$ as follows:

$$f_{i}\left(P_{n},S_{m}\right)=\left\{\begin{array}{cc} 1 & if\;D_{nmt}\geq \;A\\ 0 & others \end{array}\right.$$

If we calculate it for all images $S_{m}$ to consider the tensor $I_{n}$ compiled for each subject, we get

$$I_{n}=\left[f_{i}\left(P_{n},S_{m}\right)\right],$$

where 0≤m<M. Tensor $I_{n}$ can be expressed as a matrix that satisfies $\dim\left(Interest_{n}\right)=R^{1\times M}$.

### 3.6. Building Model for Predicting PSM

This study incorporates a mechanism to account for individual characteristics within a general gaze prediction model described in Section 3.2. The structure separates the roles of each mechanism. It enables us to narrow down the mechanisms to be focused on in gaze analysis. The study refers to the mechanism that represents individual characteristics in a vector as the user encoding mechanism.

User characteristics is a vector that reflects the unique features of each individual. The vector is essential to a model that predicts individuals’ gaze. As the user characteristics, this study adopts $I_{n}$ described in Section 3.5. Note that $I_{n}$, which reflects the interests of person $P_{n}$, is an M-dimensional vector.

Let us consider again the general gaze prediction model explained in Section 3.2. The encoder of the model extracts the characteristics from the input image using convolutional layers, while the decoder converts them into a saliency map. The encoder output is a tensor in which semantic compression has been completed for each pixel.

**Figure 4:**
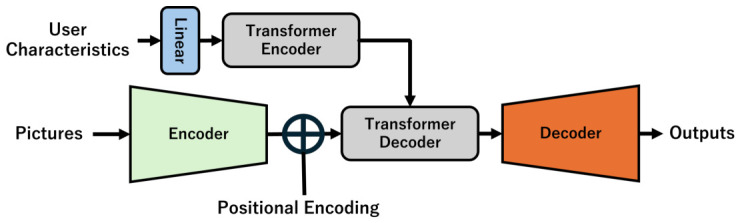
Model for predicting PSM.

Let $Enc_{out}$ be the output from the encoder, *B* be the batch size, *C* be the number of channels, and *W* and *H* be the two side lengths of the output image: $\dim(Enc_{out})$=$R^{\left(W\times H\times B\times C\right)}$. If we see the tensor per batch size, $\dim\left(Enc_{out}\right)=R^{\left(W\times H\times 1\times C\right)}$. If we regard the tensor as a one-dimensional vector, $\dim\left(Enc_{out}\right)=R^{\left((WH)\times 1\times C\right)}$. If we consider it as a two-dimensional matrix, $R^{\left((WH)\times C\right)}$, we can think of it as a set of WH pixels with *C*-dimensional elements. The *C*-dimensional vector of each pixel is the result of the compression of the information surrounding the pixel. If the dot product of the *C*-dimensional vector and a vector representing a person’s interests is large, it indicates that the person is interested in the pixel. The dot product enables us to predict each individual’s gaze. Incorporating a Transformer into a model to predict USM, this study proposes a model to predict PSMs, as shown in Figure 4.

Using the attention mechanism of the Transformer model, the model converts a pair of image features and user features into personal interests. First, consider the Transformer’s encoder. A Transformer requests that the number of channels in the input tensor of the decoder should match the number of channels in the output tensor of the encoder. In Figure 4, the output of the encoder of the model that predicts USM is connected to the input of the Transformer decoder. The number of their channels is that of $Enc_{out}$ (here, *C*). Therefore, for the Transformer decoder to work even when the output of the Transformer encoder is incorporated, the number of output channels in the Transformer encoder must be consistent with $Enc_{out}$. However, the vector $I_{n}$ representing user characteristics is *M*-dimensional, which is different from the *C*-dimensionality of the number of channels in $Enc_{out}$. Therefore, the model uses a Linear layer for a dimension transformation.

Let $T\text{\_}Enc_{in}$ be the input to the Transformer encoder, and $W_{enc}\in R^{\left(C\times m\right)}$ be the matrix that performs the linear transformation.

$$\begin{array}{c} T\text{\_}Enc_{in}=I_{n}W_{enc}^{T}\\ \dim\left(T\text{\_}Enc_{in}\right)=R^{\left(1\times C\right)} \end{array}$$

The output dimension of the encoder can have the same number of channels as $R^{\left(C\times (WH)\right)}$, which enables the inner product with $Enc_{out}$ to be calculated. Through these processes, the user characteristics are fed to the Transformer encoder. The self-attention mechanism extracts the user characteristics. The Transformer encoder provides them for the Transformer decoder as Key and Value.

Let the output from the Transformer encoder be $T\text{\_}Enc_{out}$, and the input to the first-layer Transformer decoder be $T\text{\_}Dec_{in}$. The attention Attention(Q,K,V) for head=1 can be expressed as follows:

$$\begin{array}{c} T\text{\_}Dec_{in}\in R^{\left((WH)\times C\right)},T\text{\_}Enc_{out}\in R^{\left(1\times C\right)},d_{k}=\frac{C}{head}\\ W^{Q}\in R^{\left(C\times d_{k}\right)},W^{K}\in R^{\left(C\times d_{k}\right)},W^{V}\in R^{\left(C\times d_{k}\right)}\\ Q=T\text{\_}Dec_{in}W^{Q}\\ K=T\text{\_}Enc_{out}W^{K}\\ V=T\text{\_}Enc_{out}W^{V}\\ Q\in R^{\left(\left(WH\right)\times d_{k}\right)},K\in R^{\left(1\times d_{k}\right)},V\in R^{\left(1\times d_{k}\right)}\\ Attention\left(Q,K,V\right)=softmax\left(\frac{\left(QK^{T}\right)}{\sqrt{d_{k}}}\right)V\\ Attention\left(Q,K,V\right)\in R^{\left((WH)\times C\right)} \end{array}$$

They hold regardless of the number of layers or heads in the Transformer.

The saliency map pixels whose relationship with user characteristics has been emphasized by the Transformer decoders are expected to fully encompass the interests of individual users. Finally, they are fed to the decoder to generate a saliency map. The saliency map should reflect the user’s interests.

## 4. Evaluation with Experiment

### 4.1. Experiments

To collect gaze distribution, experiments use an eye tracker, Tobii Pro Nano, connected to a laptop PC with a screen size of 1920×1080 pixels. The PC collects gaze distribution with a proprietary software tool through the eye tracker. Calibrating the eye tracker for each subject, the collection tool acquires gaze coordinates on the screen. The calibration allows more accurate acquisition of gaze distribution, eliminating physical differences between subjects.

This study involved 15 subjects, consisting of 12 men and 3 women, aged 21 to 25, with no color blindness and corrected vision. The subjects are asked to fill out a questionnaire about their hobbies. Though some subjects share common hobbies, no two subjects give the same answer. The experiments take place in a space with constant indoor lighting, to prevent the experiment environment from affecting the gaze. The subjects sitting at a desk are instructed to view 1000 images using the collection tool with periodic calibration.

The experiment uses 1000 images from the SALICON dataset. All subjects view all of these images, because different images presented to each subject would prevent us from determining whether changes in gaze are caused by individual interest or by the inherent characteristics of the image. Each subject participates in an experiment comprising 20 sets of 50 images each. Subjects take regular breaks between sets to maintain their concentration. Each experiment is conducted over one or two days, with a duration of approximately three to four hours per subject.

A total of 12,550 gaze distribution samples are obtained in the experiment. Since the sensor fails to collect the gaze for some images in certain subjects, this study analyzes 8000 samples of six men and two women, which provide sufficient gaze distribution for all 1000 images used in the experiment.

It is most desirable to collect saliency maps for many images from many users. In cases where only a small number of images are presented to each user, many users might provide a similar gaze if there are objects giving strong impressions in the images. These cases prevent from collecting personal saliency maps. Using a variety of images, this study values collecting both saliency maps common to many users and those dependent on each user to investigate what makes the differences between them. To collect data within a limited time, the study focuses on collecting data to examine the differences, with a reasonable number of subjects looking at a variety of images.

The data obtained through the eye tracker consists of gaze coordinates and pupil diameters. The study converts the gaze coordinates into a saliency map using a Gaussian filter, following the SALICON dataset [31]. Namely, the gaze coordinates for each image are smoothed using a 2D Gaussian filter with a size of 168 by 168 and a standard deviation of 24 to create a PSM. These values are set based on previous studies [31], assuming a pupil radius of 24.

### 4.2. Gaze Difference Among Subjects

If subjects with different interests present almost the same gaze for given images, it is meaningless to construct a model to predict personal interest from gaze. Let us explore the answer to the first research question: “Do individual users’ interests affect how they view each image?”

**Figure 5:**
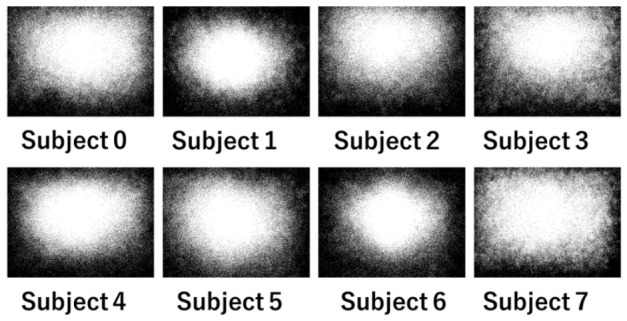
Distribution of all gaze coordinates for subjects.

Figure 5 shows, in monochrome, the distribution of all gaze coordinates for each of the eight subjects without considering the frequency of gaze occurrence at each coordinate. For all subjects, there are more gazes at the center of the image, while fewer at its edges. It is referred to as the central bias. It indicates that human gaze often converges on the center, while personal interests bring individual differences.

**Figure 6:**
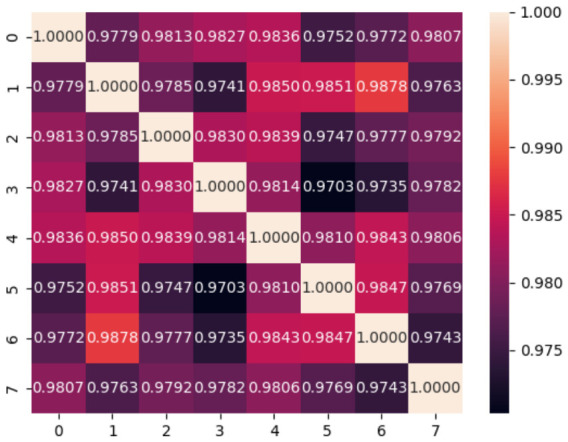
Cosine similarities between subject gaze distribution without normalization.

The differences between subjects are reflected in the cosine similarities for each subject, shown in Figure 6. The values on the vertical and horizontal axes represent each subject number. The cosine similarities for subjects 1 and 6 are particularly high, at 0.9878. We can also see that subject 4 is relatively similar to all other subjects, while subject 7 is low in similarity. However, the lowest value in Figure 6 is 0.9703, which means that the cosine similarities are high overall. The influence of the centeral bias is significant. We need to investigate whether there are any differences between subjects, excluding the center biases.

**Figure 7:**
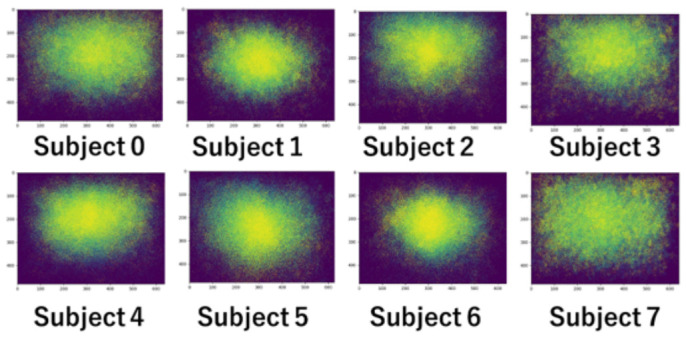
Heat map of gaze distribution without normalization.

Figure 5, which does not reflect the frequency of gaze occurrence at each coordinate, does not tell us whether gazes are concentrated in a narrow area or spread over a wide area. Let us convert the gaze information into a PSM for each subject. Figure 7 shows the gaze distribution as a heat map. As gazes are accumulated at the same coordinate, each coordinate is represented in a color ranging from deep blue to yellow. Subjects 1, 4, 5, and 6 rarely direct their gaze to the edge of the image, i.e., their gazes are mostly fixed on the center. On the other hand, the gaze of subjects 0, 2, and 3 gathers slightly above the center. Finally, subject 7 directs the gaze widely throughout the entire image.

It could come from the fact that the objects photographers intend to capture are located at the center of many photographs used in the experiments [14]. The accuracy of the model trained using such datasets would get higher if its output was closer to the center. As evaluation metrics, the study should use sAUC (Shuffled AUC) and AUC-B (AUC-Borji), which can evaluate models while reducing the effects of the central bias [12]. Table 4 shows the accuracy of the PSM calculated from gaze data collected in the experiments, expressed as sAUC and AUC-B for each subject. In the table, even sAUC, which takes center bias into account, achieves a very high evaluation of 0.877. It indicates that the PSM collected in the experiments accurately represents the tendency for gaze to converge toward the center.

On the other hand, if the collected gaze distribution does not adequately represent differences between subjects, it is difficult to create a model that predicts individual gazes. The number of gaze coordinates the tool collects from each image is fixed to 300. As a result, the gaze distribution varies with subjects; the number of gazes around the center gets small, while the area where gazes gather will differ for each subject. It reduces central bias, which allows us to investigate individual gazes, including the influence of the unique characteristics of each image.

**Figure 8:**
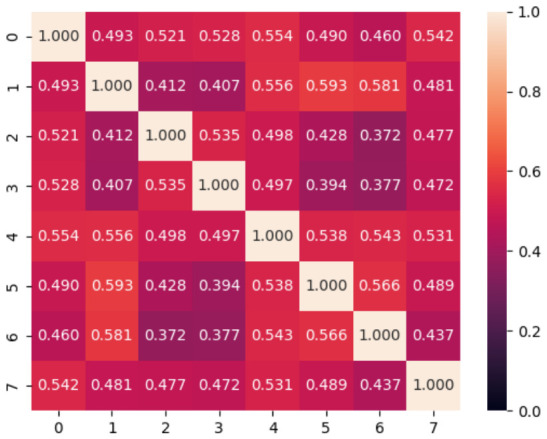
Cosine similarity between subject PSMs.

Let us compare PSMs between subjects. Figure 8 shows the average cosine similarity between subject PSMs. The differences can be regarded as the one in gaze patterns between subjects. Compared to Figure 6, the PSM similarity in Figure 8 is low. Subjects 1, 4, 5, and 6, who have high cosine similarity in Figure 6, also had relatively high cosine similarity in Figure 8. It indicates that subjects tend to fixate on the same object, that is, they exhibit similar gaze movements even when the influence of the central bias is suppressed. However, the maximum cosine similarity and the minimum are 0.593 and 0.372, respectively. The values are low as a whole.

The PSMs show high similarity due to the central bias, but they also present differences in gaze tendencies between individuals for each image. When humans are interested, they focus their gaze on the object of interest [6]. In answer to the first research question, we can say that differences in interest affect gaze. However, note that the differences among subjects are extremely small, with a maximum of 0.221. It would be undesirable to use PSMs as labels for training a gaze prediction model. We should prepare user feature vectors to be used for the labels.

### 4.3. Change of Pupil Diameters

**Figure 9:**
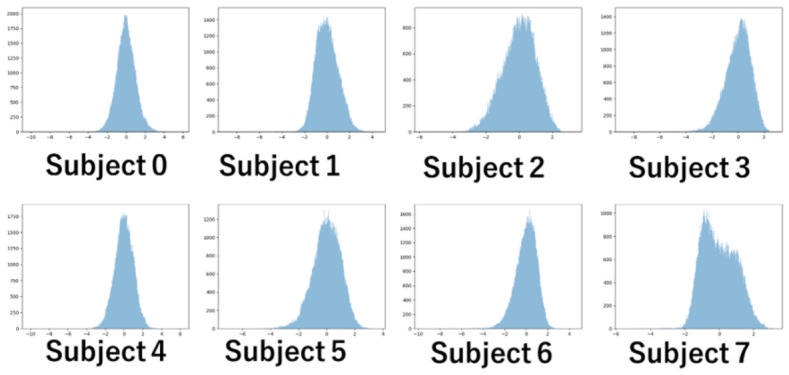
Histogram of subject pupil diameters.

The experiments record changes in pupil diameter over time for each image. Figure 9 shows a histogram of the pupil diameter for each subject after the standardization. If personal interests change the pupil diameter, the gaze distribution will differ from a Gaussian distribution, which represents randomness. It should result in gazes gathering in areas other than the average.

**Figure 10:**
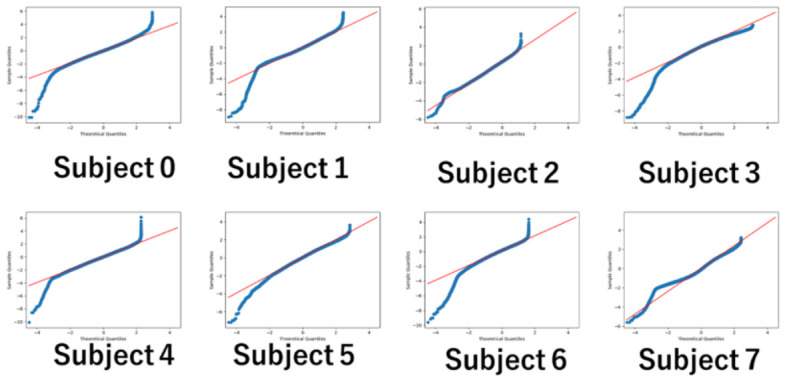
QQ plots of subject pupil diameters.

Figure 10 shows QQ plots of the samples in Figure 9. The red line represents the normal distribution. For most subjects, both ends of the distribution deviate significantly from normality. It indicates that a significant number of samples are distributed far from the average.

To demonstrate that the pupil diameter distribution deviates from normality, an anomaly test based on the chi-squared distribution is applied to extract both sides of the distribution. Two significance levels, 1% and 10%, are used to determine which is more effective in extracting pupil diameters that deviate from normality. It allows us to determine the degree of their deviation suitable to detect personal interests.

**Figure 11:**
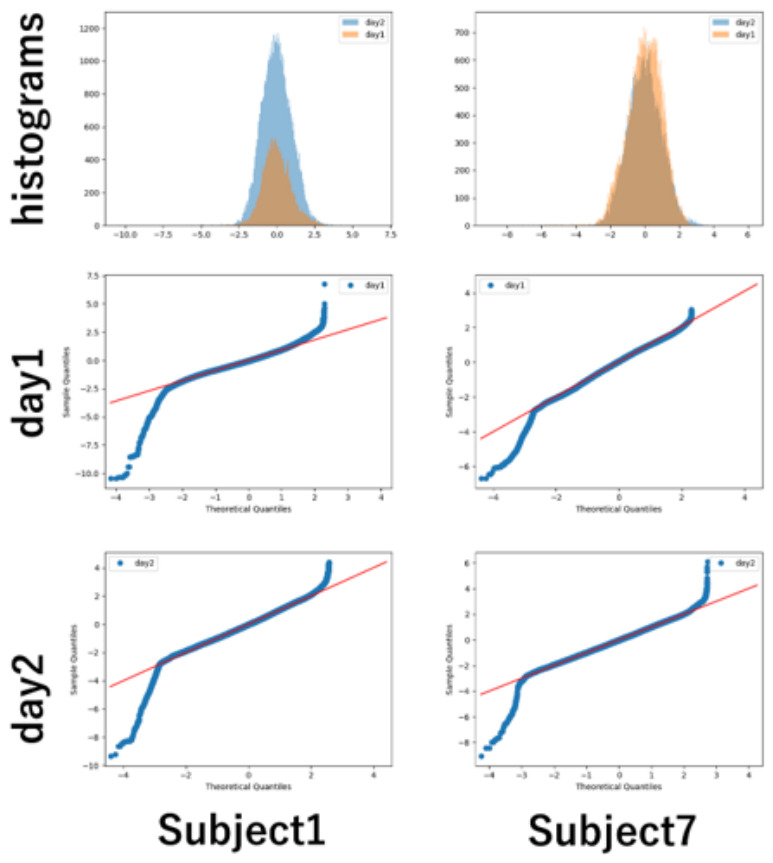
Histograms and QQ plots of subject 1 and 7 for each day.

The QQ plot for subject 7 has an unusual shape compared to the other subjects. It has two centers. It could be due to the difference in the number of days spent on the experiment. While most subjects complete their experiments in one day, subjects 1 and 7 take two days. Figure 11 shows the pupil diameters of subjects 1 and 7, standardized by experiment day, in a histogram and QQ plot. They have the same shape as the distribution for the other subjects. Limited to subjects 1 and 7, the anomaly testing is applied to samples of each day to extract thresholds for their personal interests.

**Figure 12:**
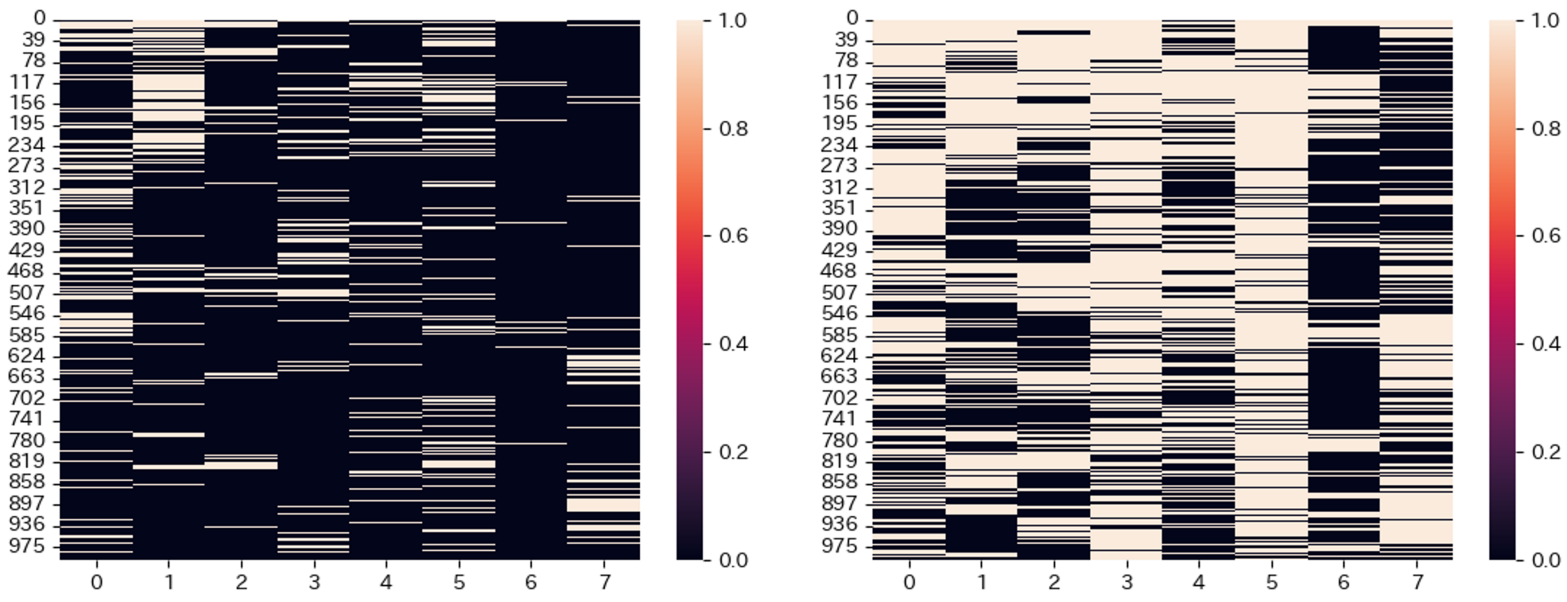
Pupil dilation of each subject per image.

Figure 12 shows the pupil dilations for every subject at each significance level. The left and right graphs show the 1% significance level and 10% significance level, respectively. The vertical axis shows the identifiers of the 1000 experimental images, while the horizontal axis shows the identifiers of each subject. In the graphs, if the pupil dilation is greater than the significance level for the image, it is depicted with the bright color, and otherwise with the dark color. From the 1% significance level graph, we can see that pupil dilation depends on each subject. Greater pupil dilations are not uniformly distributed across all subjects. Some subjects barely present them. The bright areas of the 10% significance level cover those of the 1% significance level. However, even at the 10% significance level, there are images for which few subjects present pupil dilation.

The gaze collection tool used in the experiment measures pupil diameter approximately 300 times over 5 s. If pupil diameter changes randomly, regardless of the image, pupil dilation should be observed in almost all images. However, it is not the case in Figure 12. It suggests that there is some factor behind the change in pupil diameter. It has been shown that human pupil size varies with interest [32]. The pupil diameter is enlarged due to the subjects’ interest in the image’s characteristics. The paper regards the pupil dilation for each image as a user characteristic vector, which represents the interests of each subject.

**Figure 13:**
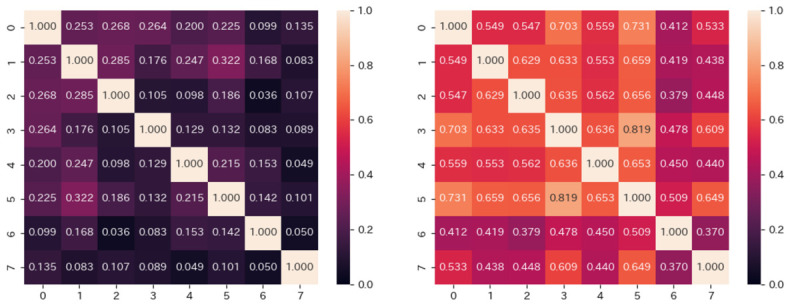
Cosine similarity of each subject’s interest (**Left**: 1%, **Right**: 10%).

Figure 13 shows the cosine similarity of interest for every pair of subjects at each significance level. The values on the vertical and horizontal axes represent each subject. Subjects 1 and 5 have high cosine similarity. It indicates that subjects 1 and 5 are often similar in interests, compared to other subjects. On the other hand, at a significance level of 1%, subject 4, whose gaze distribution is similar to those of the other subjects in Figure 8, has a low cosine similarity with the other subjects. While subject 4 is similar to the other subjects in gaze distribution, the images that interest him are different from those of the other subjects. A similar gaze distribution does not necessarily mean a similarity in interest.

As shown in Figure 12, when the significance level is set to 10%, subjects 3 and 5 judge most images as interesting. Their cosine similarity is 0.819, indicating a high similarity. On the other hand, when the significance level is set to 1%, there are few images judged as interesting by both subjects 3 and 5. Their cosine similarity is 0.132, indicating that they are no longer similar to each other. For all subjects, when the significance level is changed from 1% to 10%, the cosine similarity increases to a high value. The increase also occurs for subject 7, whose cosine similarity is low with all other subjects.

Indeed, user feature vectors at a significance level of 1% would strictly detect interest, but the strict detection of pupil diameters causes a risk of missing cases where the subject is interested. Actually, as shown in Figure 12, when the significance level is set to 1%, most subjects find no images interesting. It makes no sense. On the other hand, as Figure 12 shows, each subject presents individual interests in images when the significance level is set to 10%. It is plausible to adopt a 10% significance level to detect changes in pupil diameter that correspond to interest. Here, we should note that the detection of the significance level at 10% accounts for pupil diameter dilation due to image characteristics, which commonly affects many people.

### 4.4. A Model for Predicting General Gaze

A general gaze is a distribution of gazes that appear commonly among many people for a given image. Let us build a model to predict it. This study uses the SALICON dataset. Of the 10,000 training images, 1000 have been used to identify user feature vectors and predict gazes. Using the remaining 9000 images, a model is trained to predict general gazes. To reduce the model size and training costs, the input images are compressed to one-quarter their original size. As a result, the output images are also one-quarter the size.

The batch size during training is 32. The optimizer is Adam, with the learning rate $1.0\times 10^{-5}$. To prevent overfitting, early stopping will take place if the loss on the validation data does not decrease for five consecutive iterations.

Table 5 shows the model’s performance, calculated on validation data from the SALICON dataset. The indices NSS and CC represent the saliency of gaze coordinates and the correlation between the predicted results and the ground truth map, respectively. Table 5 shows that the model’s performance is comparable to that of SalGAN. It demonstrates sufficient gaze prediction. The results suggest that the model’s parameters adequately represent general gaze characteristics. This study trains a model to predict individual gaze, setting the parameter values as its initial values.

### 4.5. A Model for Predicting Personal Gaze

The model for predicting individual gaze takes two inputs: the user feature vector and the image used to create the PSM. These inputs produce an output. The model is trained to reduce the output error from the PSM corresponding to the model’s input image. This study makes a set of two inputs and one output to train the model with a total of 8000 sets of data. The model has the same encoder and decoder structure as the general gaze prediction model, which enables the parameters of a general gaze prediction model to be directly substituted for those of the trained model.

Of the total 8000 datasets, 6400 are used for training and 1600 for validation. As with general gaze prediction models, the input images and PSM are compressed to 1/4 their original size, and the output size is also set to 1/4. The user characteristic vector at a significance level of 10% is labeled as 1. The training data and validation data are divided so that the number of images labeled with 1 is uniform for each subject.

Based on CvT [28], a model that incorporates CNN into a Transformer, the study adopts AdamW as the optimizer, with a weight of 0.05, and a learning rate of $1.0\times 10^{-6}$. The model trains each image eight times within one epoch. For this reason, when calculating the average for each batch size, certain image characteristics become more pronounced. Therefore, the batch size is set to 1. As with general gaze prediction models, early stopping is applied if the loss on the validation data does not decrease for five consecutive times.

The Transformer model used in the user encoding mechanism is divided into a Transformer encoder and a Transformer decoder. Each of these can have any number of layers. It is necessary to determine the optimal combination of the number of layers for the Transformer encoder and Transformer decoder. Furthermore, the number of heads can be changed to account for multi-head attenuation in the Transformer model. This study first fixes the number of heads to 32 and the user feature vector at a significance level of 10% to determine the optimal combination of layers for the Transformer encoder and Transformer decoder that minimizes the error of the validation data. If the parameters of the model predicting general gaze are used as initial values, the influence of the mechanism encoding user features gets weak, which makes it difficult to observe differences in the number of Transformer layers. The study does not use the parameters of the model predicting general gaze to examine the number of layers.

Table 6 shows the number of layers and the corresponding loss function output. MSE is used as the loss function. Since the batch size is 1, the values in the table are simply the average MSE for all 1600 validation data. Experimental results show that the error is minimized when the Transformer encoder and decoder have four and nine layers, respectively. The self-attention translates the user feature vector, which is input to the Transformer encoder. The optimal number of layers differs between the Transformer encoder and the Transformer decoder because the more layers there are, the less the user feature vector represents the characteristics of the image features. It is necessary to limit the number of layers in the Transformer encoder so that the user feature vector can represent the unique relationship of each individual with each pixel in the image.

Next, let us examine the effect of the number of multi-heads. Setting the Transformer encoder to four layers and the Transformer decoder to nine layers, the examination changes the number of heads to find the number that minimizes the error. The examination incorporates the parameters of a general gaze model into the learning process. It keeps the number of layers in the user encoding mechanism, as well as the encoder and decoder sections, in their optimal states, allowing us to investigate the effect of pure multi-heads.

Table 7 shows the results of the examination when the significance level for setting user feature vectors is 1% and 10%, and the number of heads for the Transformer encoder and Transformer decoder is changed to 32, 64, 128, and 256. The table shows that the error is minimized when the significance level for creating user feature vectors is 10%, and the number of heads is 256. Furthermore, as the number of heads increases, a gradual decrease in error can be observed in most cases. Let us add a case where the significance level is 10%, and the number of heads is 512. The table shows that the error increases when the number of heads is 512. It might occur because the number of heads assigned to each CNN filter in the encoder section decreases as the number of heads increases. When the number of heads is 512, one head is assigned to each filter in the final layer, which means that the Transformer’s Query, Key, and Value become one-dimensional, making it difficult to represent individual characteristics properly.

The above shows that each element has a unique meaning in the user feature vector at a significance level of 10%. By paying attention to each element, the vector can clarify differences in individual subjects. However, it is interesting to note that the error is smaller at a significance level of 10% than at a significance level of 1%, and that the error increases when we pay attention to too few elements. At a significance level of 10%, attention includes factors common to more subjects. Factors are represented by multiple elements of the vector. If the elements are separated one by one, the vectors cannot fully represent the differences between subjects. It is consistent with the idea that we have better to consider image characteristics common to many people to detect pupil diameter dilation, as explained in Section 4.3. User feature vectors should be designed with the influence of general image characteristics in mind.

Based on the above examination, the optimal model for predicting an individual’s gaze should use a user characteristic vector obtained at a significance level of 10%, taking into account the influence of general image characteristics. The model should have four layers of Transformer encoder, nine layers of Transformer decoder, and 256 heads.

Table 8 shows the performance of the model for predicting personal gaze at optimal parameter settings. Subject 7 has the lowest sAUC of 0.7145, while the average is 0.7758. The model can express the gaze of each subject. Among the subjects, the model has the highest prediction for subjects 1 and 6.

**Figure 14:**
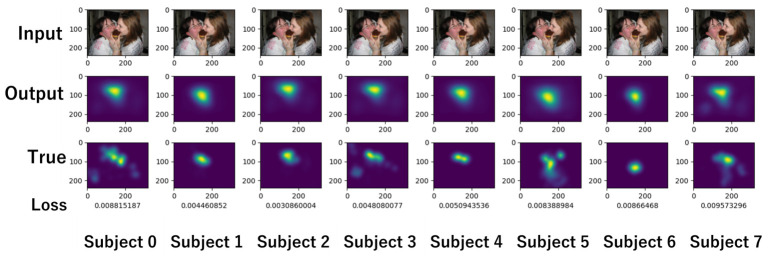
Outputs of model for predicting personal gaze.

Figure 14 shows an example output. The top row shows the input image, the middle row shows the model prediction results, and the bottom row shows the saliency map obtained from the subject’s gaze. Below the saliency map are the loss values. It can be seen that the model output tracks the differences in the position and spread of the saliency map for each subject.

## 5. Relationship Between General Gaze and Individual Gaze

Section 4 has revealed that predicting individual gaze distribution requires an optimal model structure and a user characteristic vector that includes not only individual characteristics but also factors common to many subjects. However, it has not investigated the reasons why they are useful for predicting individual gaze distribution. This section uses the results of Section 4 to discuss the elements necessary for predicting individual gaze distribution.

A single USM is attained, aggregating the gaze data of multiple subjects for the SALICON dataset. The parameters of the model trained to predict the USM sufficiently reflect information about general gaze. Through analyzing how these parameters affect the prediction of individual gaze, we can clarify how differences between general and individual gaze arise.

The previous section examines the performance of the training model, both with and without the parameters for general gaze prediction as initial values. It turns out that training incorporating a general gaze prediction mechanism is more successful in making predictions that reflect individual differences between subjects. However, to understand the effect of the parameters, we needed to visualize the internal state of the model.

### 5.1. Model to Verify Hypothesis

**Figure 15:**
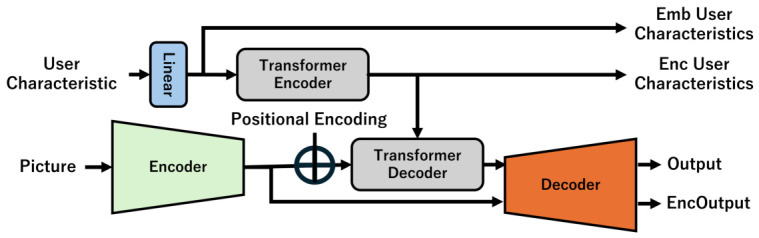
Model to verify hypothesis.

To predict USM, which is a general gaze, this study trains a mechanism comprising an encoder that maps an input image to a code and a decoder that reconstructs the code into an image. As shown in Figure 15, the study adds a user encoding mechanism to the model to incorporate each user’s characteristics. The configuration is adopted because the study assumes that an individual’s gaze is a variation of a general gaze.

The Linear layer of the user encoding mechanism embeds the user characteristics so that they can be fed to the Transformer layer. EmbUserCharacteristics is the user characteristic vector mapped by the Linear layer. EncUserCharacteristics is the user characteristic vector after being transformed by the Transformer encoder layer. Rather than training a model from scratch, its initial values are set to the trained parameters of a model that predicts USM. This paper discusses how the learning progresses, examining the output of each mechanism.

The Enc Output of the model is an image generated by the decoder from the encoder output, without passing it through the user encoding mechanism. By comparing the Enc Output with the inherent output, we can visualize the impact of the user encoding mechanism on gaze prediction. The paper compares them when the parameters of the model that predicts USM are used as the initial values during training, and when they are not. It investigates the impact on gaze prediction

### 5.2. Training Independent of USM Parameters

**Figure 16:**
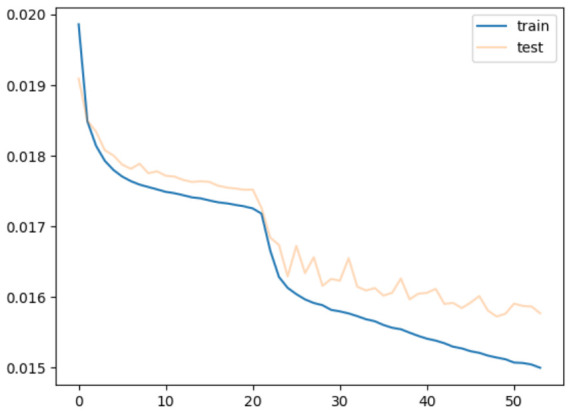
Training from random initial values.

Figure 16 shows the learning curve for the model trained with parameters of the encoder and decoder initialized with random values. It is a training using the encoder and decoder independent of the USM. The horizontal axis represents the number of epochs, while the vertical axis represents the error. In this study, if the lowest error value for the validation data is not updated for five successive times, early stopping is used to stop training. In the experiment, the learning stopped at 53 epochs. The figure indicates that the error for both the training and the validation data drops significantly around 20 epochs.

**Figure 17:**
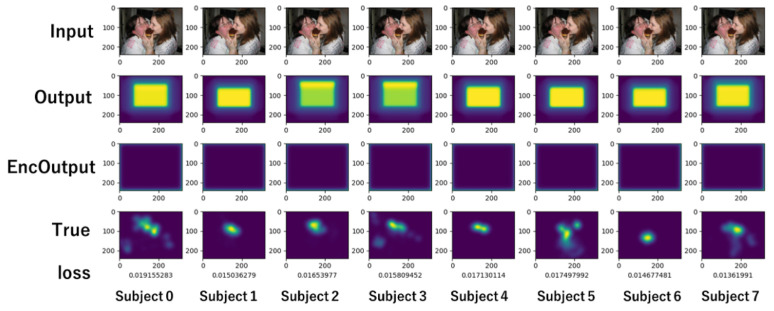
Visualization at 20 epochs.

**Figure 18:**
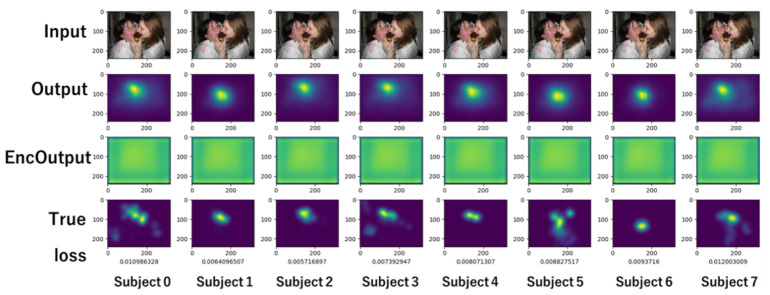
Visualization at 53 epochs.

Figure 17 is a visualization at 20 epochs, while Figure 18 is one at the end of the learning. In both figures, from top to bottom, the input image, predicted gaze, visualized Enc Output, and actual gaze collected from the subjects are arranged for eight subjects. The visualization in Figure 18 indicates that each subject’s gaze is predicted to some extent, because the output follows the actual gaze in each subject.

The pixels in EncOutput are almost 0 in Figure 17. The result indicates that the encoder has rarely learned at the 20th epoch point. However, the output for each subject does not provide 0, but a monotonous rectangle. It is different for each subject. In terms of deviation from the center, the output is coincident with the actual gaze indicated by the “True” label. In Figure 18, we can see that Enc Output provides high values widely across the entire image. The figure also shows that the output is more consistent with the actual gaze distribution of each subject than that of Figure 17. Since the gaze distribution of each subject is the subject’s common gaze over many images, it presents a gaze tendency unique to the subject. It means the user encoding mechanism learns each subject’s unique gaze tendency earlier than the other parts. The results imply that the user encoding mechanism alone predicts an individual subject’s gaze using each subject’s unique gaze tendency in the early stage of the learning where the model is trained independently of USM parameters.

### 5.3. Training Based on USM Parameters

**Figure 19:**
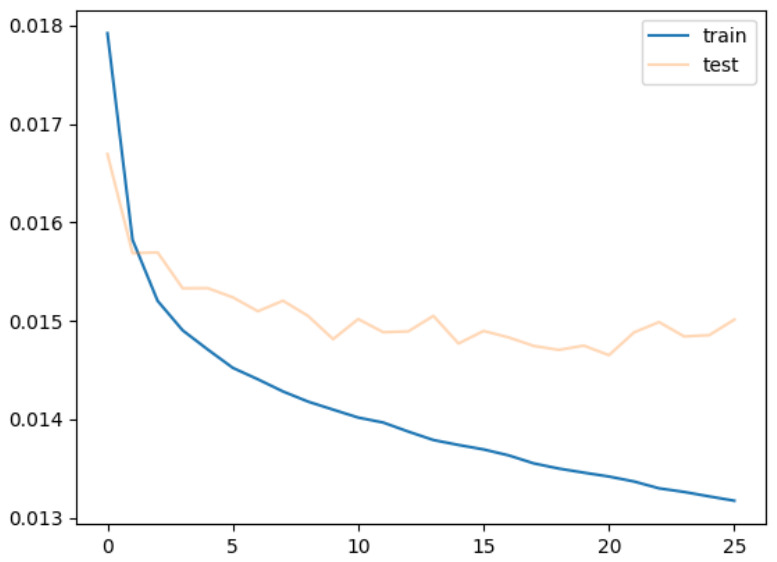
Training based on USM parameters.

Figure 19 shows the learning curve of the model when the decoder and encoder parameters that predict USM are given as initial values. The learning ends after epoch 25. The learning finishes earlier than the training using parameters independent of USM.

**Figure 20:**
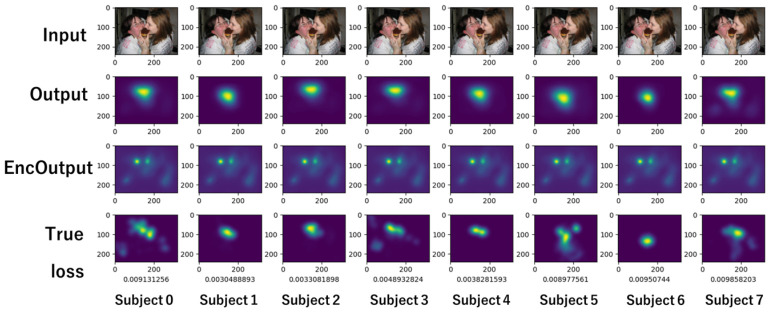
Visualization of training results based on USM parameters.

Figure 20 is a visualization of the training results based on USM parameters. Compared to Figure 18, which shows the training results independent of USM parameters, EncOutput is significantly different.

**Figure 21:**
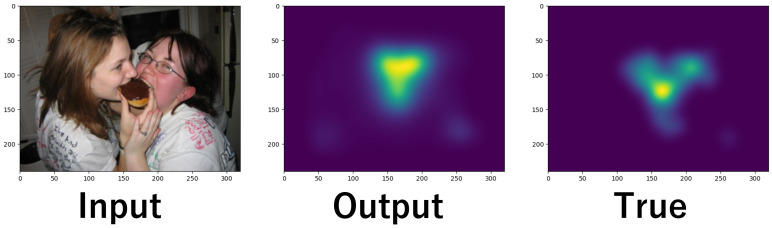
Visualization of model for USM.

Figure 21 is a visualization of a model that predicts USM. The model parameters are reused for predicting PSMs. The model proposed in this study predicts gaze for each individual by adding a user encoding mechanism. In Figure 20, the output has a shape made from the gaze distribution of the USM. It can be assumed that the model learns to predict individual gaze distribution, altering the given USM as a reference.

### 5.4. Predictability of PSM with Gaze

The second research question of the paper asks whether personal interests can be predicted by gaze.

Let us examine how much difference the USM parameters bring to vectors representing characteristics of each subject. Table 9 shows the cosign similarity of EmbUserCharacteristics with and without the USM parameters, while Table 10 shows that of EncUserCharacteristics, respectively. EmbUserCharacteristics do not change significantly with USM parameters, but there is a large difference in EncUserCharacteristics converted by the Transformer encoder layer. The information required by each mechanism differs depending on whether USM parameters are used as the initial values. It might come from the foundation of the learning. Learning independent of USM parameters is founded on the commonality of each subject’s gaze across images, whereas learning based on USM parameters is founded on similarity to USM.

**Figure 22:**
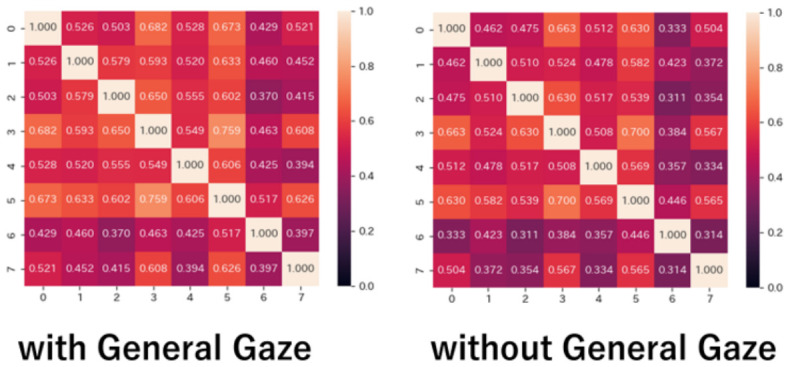
Cosine similarity between each subject in EncUserCharacteristics.

Figure 22 shows the cosine similarity between subjects for EncUserCharacteristics. There is not much difference in the similarity. For example, subjects 3 and 5 are highly similar, while subjects 2 and 6 are low in similarity. The user characteristic vector used as input has 800 dimensions, while EncUserCharacteristics has 512 dimensions, the same as the input dimensions of the Transformer decoder layer. The Transformer encoder extracts information from the inputs. The two vectors of EncUserCharacteristics based on/independent of USM parameters are coincident in subject similarity because the Transformer encoder succeeds in extracting interest differences between subjects.

The results show that the model is trained to represent differences in user characteristic vectors, regardless of whether USM parameters are used. We can predict personal interests with gazes, which is the answer to the second research question.

### 5.5. Requisites for Predictability of PSM

Naturally, pupil diameter will expand not only when each individual has a unique interest, but also when there is an object that everyone is paying attention to. The user characteristic vector in the study represents pupil diameter expansion due to each subject’s interest, as well as pupil diameter expansion due to image characteristics that attract the attention of many people.

Within an image, individual gazes are drawn to objects matching the individual’s interest factors, starting from objects attracting many people. The output of the encoder, which predicts common gazes among many people, picks up objects that may be interesting to the subject. This study regards individual gazes as variations of gazes common to many people. Based on the person’s gaze tendencies, the variations arise according to characteristic objects in the image. It is explained by the change in the output of the model independent of USM parameters; it is first individual gaze tendencies, and then changes according to each image. Individual differences in gaze do not arise solely from interesting objects specific to individual interests within an image. It is reasonable to consider that they arise when objects attracting many people get the subject’s unique interests. As stated in Section 4.5, the user characteristic vector should include not only individual-specific characteristics but also the influence of general image characteristics.

Training with USM parameters as initial values corresponds to alternation of the initial position of learning. The state of learning is represented by a set of weights to be trained. The USM parameters affect the learning process because the learning proceeds through the error space along different paths depending on the initial position. If the initial position is located in a location far from the USM, the model takes a path in which it first attains the individual’s gaze tendency, next following the path to reach the USM. The fact that models reach similar results even in different paths indicates that the point of the smallest error is located in a certain area in the error space. The area passed during learning to attain the individual’s gaze tendency might partially overlap that passed during learning to attain the USM. The results suggest that the optimal point for PSM might be located in a hyperspace between an area for USM and one for an individual’s gaze tendency. When a model has mechanisms that represent the two areas, we can predict PSM.

Based on the above discussion, the model proposed in this study for individual gaze prediction requires the following four elements:

- Mechanism that can predict general gaze;
- Mechanism that can predict personal gaze;
- A training dataset containing common gaze patterns among many people;
- A training dataset containing individual gaze characteristics.

It is the answer to the third research question. Note that a single dataset may contain both of the last two items.

## 6. Advantages of Proposed Method

**Figure 23:**
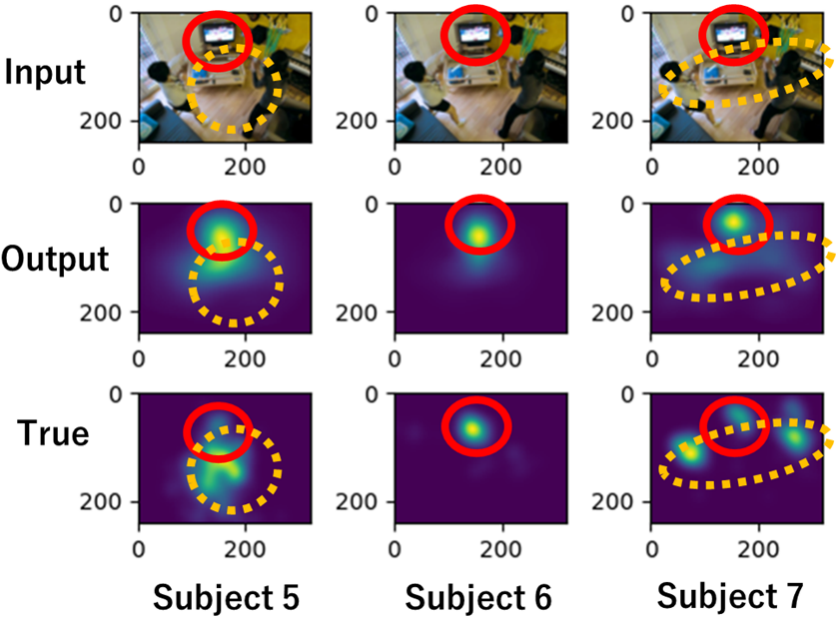
Common areas and personally different areas in predicted SM.

The proposed model predicts both USM and PSM. Figure 23 shows the SMs for subjects 5, 6, and 7 on the same image. The area enclosed by the red frame contains a television, which attracts the attention of all subjects. Since the model predicts individual gaze patterns based on general gaze patterns, it is reflected in the prediction results. The common predicted areas allow us to identify locations that generally attract human attention. For example, we can apply the model to suggest placement locations for advertisements. On the other hand, the yellow frame represents predicted locations individually different in gaze patterns. The results enable us to identify personalized recommendations and analyze the factors influencing individual interests.

Some models for predicting PSM use USM as input [33,34]. However, their performance depends on the accuracy of the USM. Furthermore, in these methods, it is necessary to build a USM each time to predict PSM, which is costly. PSM should be predicted only with an image and user information.

The proposed method constructs a model capable of predicting PSM through training based on a pre-trained USM model. The method can predict PSM even for images without a USM.

This study determines user characteristic vectors representing user interest, using pupil dilation as an indicator. Another indicator representing user interest is microsaccades [35,36]. While saccades are rapid, voluntary eye movements when scanning the surroundings, microsaccades are involuntary, minute eye movements that occur when staring at a single point. Microsaccades caused by focusing on an object enable us to identify a user’s areas of interest.

The proposed model in the paper does not limit the method for creating user characteristic vectors to a specific method. Vectors created with indicators that more faithfully reflect individual user interests, such as microsaccades in addition to pupil diameter, are expected to improve the accuracy of PSM prediction.

## 7. Conclusions

This study proposes a method for predicting individual interests from their gaze. To predict gaze that reflects each individual’s interests, it builds a model for predicting PSMs. Experimental results have shown that, to express each individual’s gaze, it is necessary to combine two different mechanisms with different roles: a mechanism for predicting general gaze and one for expressing each individual’s gaze tendencies. The results also reveal that we should use two different characteristics: characteristics that represent general gaze and those that represent differences in gaze for each individual. The results of the study enable us to predict each individual’s PSM, which leads to objective identification of the interests of each individual.

This study examines only eight subjects. It has not been verified whether the model works for all individual characteristics. When adding a new person to the prediction target, the proposed method requires making a user characteristic vector. The vector is dependent on individual interests in the images used in the experiment. As a result, when adding a new subject, it is necessary to record gaze and pupil diameter. It poses the problem of high implementation costs per subject. To reduce the cost of applying the model, it is necessary to verify whether the model works consistently with vectors representing interest factors extracted using other methods with low implementation costs. 

## Figures and Tables

**Table 1:** Layers of encoder.

Layer	Depth	Kernel	Stride	Padding	Activation
Conv2D	64	1*1	1	1	ReLU
Conv2D	64	3*3	1	1	ReLU
MaxPool2D		2*2	2	0	
Conv2D	128	3*3	1	1	ReLU
Conv2D	128	3*3	1	1	ReLU
MaxPool2D		2*2	2	0	
Conv2D	256	3*3	1	1	ReLU
Conv2D	256	3*3	1	1	ReLU
Conv2D	256	3*3	1	1	ReLU
MaxPool2D		2*2	2	0	
Conv2D	512	3*3	1	1	ReLU
Conv2D	512	3*3	1	1	ReLU
Conv2D	512	3*3	1	1	ReLU
MaxPool2D		2*2	2	0	
Conv2D	512	3*3	1	1	ReLU
Conv2D	512	3*3	1	1	ReLU
Conv2D	512	3*3	1	1	ReLU

**Table 2:** Layers of decoder.

Layer	Depth	Kernel	Stride	Padding	Activation
Conv2D	512	3*3	1	1	ReLU
Conv2D	512	3*3	1	1	ReLU
Conv2D	512	3*3	1	1	ReLU
UpSample		2*2	2	0	
Conv2D	512	3*3	1	1	ReLU
Conv2D	512	3*3	1	1	ReLU
Conv2D	512	3*3	1	1	ReLU
UpSample		2*2	2	0	
Conv2D	256	3*3	1	1	ReLU
Conv2D	256	3*3	1	1	ReLU
Conv2D	256	3*3	1	1	ReLU
UpSample		2*2	2	0	
Conv2D	128	3*3	1	1	ReLU
Conv2D	128	3*3	1	1	ReLU
UpSample		2*2	2	0	
Conv2D	64	3*3	1	1	ReLU
Conv2D	64	3*3	1	1	ReLU
Conv2D	1	1*1	1	0	ReLU

**Table 3:** Collected data items.

Data Item	Explanation
Timestamp	Elapsed Seconds from Image Presentation
Left Eye Gaze Coordinates	Coordinates based on the top left
Right Eye Gaze Coordinates	Coordinates based on the top left
Left Pupil Diameter	Left Eye Pupil Size
Right Pupil Diameter	Right Eye Pupil Size

**Table 4:** Accuracy of PSM.

Subject ID	sAUC	AUC-B
0	0.9071156618680438	0.9419253886144336
1	0.9158976654517679	0.9641849007734729
2	0.9045685597027522	0.9468337457837294
3	0.9176894376891533	0.9469340783127931
4	0.8994813998914786	0.9462664041017854
5	0.8773627780413019	0.9286451929073957
6	0.9045921468163373	0.9553466929167967
7	0.9410767264429488	0.959907013306794
mean	0.908473046987973	0.94875542708965

**Table 5:** Performance of model to predict general gaze.

sAUC	AUC-B	NSS	CC
0.8556327903801951	0.8622561397074704	1.707360242253475	0.8399891614959102

**Table 6:** Optimal layer numbers of encoder and decoder of Transformer.

Enc/Dec	MSE Loss
3/6	0.016044
3/9	0.016149
4/8	0.015959
4/9	**0.015803**
4/12	0.017498
6/6	0.016218
9/9	0.01603

**Table 7:** Optimal number of heads.

Type/Heads	MSE Loss
1%/32	0.014936686
1%/64	0.014898729
1%/128	0.014857693
1%/256	0.014764848
10%/32	0.015030035
10%/64	0.01494549
10%/128	0.0149971135
10%/256	**0.0147023015**
10%/512	0.014754556

**Table 8:** Performance of model for predicting individual gaze.

Subject	sAUC	AUC-B	NSS	CC
0	0.7500	0.7657	1.0255	0.4372
1	0.8312	0.8630	1.6853	0.5729
2	0.7534	0.7834	1.1055	0.4839
3	0.7443	0.7580	0.9887	0.4195
4	0.7912	0.8147	1.2609	0.5251
5	0.7968	0.8146	1.3225	0.5946
6	0.8254	0.8535	1.7067	0.5282
7	0.7145	0.7357	0.8504	0.3479
mean	0.7758	0.7986	1.2432	0.4887

**Table 9:** Cosine similarity of EmbUserCharacteristics with/without USM parameters.

Subject	Cosine Similarity
0	0.9148947
1	0.95943834
2	0.94265401
3	0.92215813
4	0.93176272
5	0.93883808
6	0.97130742
7	0.94247958
mean	0.9404416225

**Table 10:** Cosine similarity of EncUserCharacteristics with/without USM parameters.

Subject	Cosine Similarity
0	0.40764675
1	0.71327605
2	0.45038623
3	0.41545308
4	0.49677647
5	0.70966673
6	0.75337164
7	0.45110928
mean	0.5497107787500001

## Data Availability

No new data were created or analyzed in this study.
